# Increasing multimonth dispensing of antiretrovirals and assessing the effect on viral load suppression among children and adolescents receiving HIV services in Nigeria

**DOI:** 10.1371/journal.pone.0286303

**Published:** 2023-06-14

**Authors:** Caterina Casalini, Moses Bateganya, Chris Akolo, Olusola Sanwo, Augustine Idemudia, Pius Nwaokoro, Frank Eyam, Matthew-David Ogbechie, Chika Obiora-Okafo, Abimbola Oduola, Rose Wilcher, Natasha Mack, Hadiza Khamofu, Satish Raj Pandey

**Affiliations:** 1 FHI 360, Hilversum, Netherlands; 2 FHI 360, Washington, DC, United States of America; 3 FHI 360, Abuja, Nigeria; 4 FHI 360, Papua New Guinea; 5 FHI 360, Durham, NC, United States of America; Georgetown Global Health Nigeria, NIGERIA

## Abstract

**Introduction:**

Multimonth dispensing (MMD) enables less frequent clinic visits and improved outcomes for people living with HIV, but few children and adolescents living with HIV (CALHIV) are on MMD. At the end of the October–December 2019 quarter, only 23% of CALHIV receiving antiretroviral therapy (ART) through SIDHAS project sites in Akwa Ibom and Cross River states, Nigeria, were receiving MMD. In March 2020, during COVID-19, the government expanded MMD eligibility to include children and recommended rapid implementation to minimize clinic visits. SIDHAS provided technical assistance to 36 “high-volume” facilities—≥5 CALHIV on treatment—in Akwa Ibom and Cross River to increase MMD and viral load suppression (VLS) among CALHIV, toward PEPFAR’s 80% benchmark for people currently on ART. We present change in MMD, viral load (VL) testing coverage, VLS, optimized regimen coverage, and community-based ART group enrollment among CALHIV from the October–December 2019 quarter (baseline) to January–March 2021 (endline) based on retrospective analysis of routinely collected program data.

**Materials and methods:**

We compared MMD coverage (primary objective), and optimized regimen coverage, community-based ART group enrollment, VL testing coverage, and VLS (secondary objectives), among CALHIV 18 years and younger pre-/post-intervention (baseline/endline) at the 36 facilities. We excluded children younger than two years, who are not recommended for or routinely offered MMD. The extracted data included age, sex, ART regimen, months of ART dispensed at last refill, most recent VL test results, and community ART group enrollment. Data on MMD—three or more months of ARVs dispensed at one time—were disaggregated into three to five months (3–5-MMD) vs. six or more months (6-MMD). VLS was defined as ≤1,000 copies. We documented MMD coverage by site, optimized regimen, and VL testing and suppression. Using descriptive statistics, we summarized the characteristics of CALHIV on MMD and non-MMD, number of CALHIV on optimized regimens, and proportion enrolled in differentiated service delivery models and community-based ART refill groups. For the intervention, SIDHAS technical assistance was data driven: weekly data analysis/review, site-prioritization scoring, provider mentoring, line listing eligible CALHIV, pediatric regimen calculator, child-optimized regimen transitioning, and community ART models.

**Results:**

The proportion of CALHIV ages 2–18 receiving MMD increased from 23% (620/2,647; baseline) to 88% (3,992/4,541; endline), while the proportion of sites reporting suboptimal MMD coverage among CALHIV (<80%) decreased (100% to 28%). In March 2021, 49% of CALHIV were receiving 3–5-MMD and 39% 6-MMD. In October–December 2019, 17%–28% of CALHIV were receiving MMD; by January–March 2021, 99% of those 15–18 years, 94% 10–14 years, 79% 5–9 years, and 71% 2–4 years were on MMD. VL testing coverage remained high (90%), while VLS increased (64% to 92%). The proportion on pediatric-optimized regimens increased (58% to 79%).

**Conclusions:**

MMD was feasible among CALHIV without compromising VLS. Expanded eligibility criteria, line listing eligible children, monitoring pediatric antiretroviral stock, and data use contributed to positive results. Future efforts should address low 6-MMD uptake related to stock limitations and synchronize antiretroviral refill pickup with VL sample collection.

## Introduction

Globally, an estimated 28.2 million people living with HIV (PLHIV) (74% of adults and 54% of children) [1] were on life saving antiretroviral therapy (ART) as of June 2021, up from 17.1 million in 2015 when test and treat was first recommended by the World Health Organization (WHO) [2]. With the growing number of patients benefiting from test and treat, donors, policymakers, and program implementers are looking for efficient service delivery models that reduce the burden for patients and health systems. Long waiting times in ART clinics, cost of travel to and from clinics, missed wages as result of being away from work, and other life commitments can disrupt and reduce adherence and retention among ART clients [3]. Innovative systems that require less frequent facility visits for refills and clinical assessments may result in fewer treatment interruptions [4] and could improve clinical outcomes such as viral load suppression (VLS)—which, in 2020, was only 40% among children 0–15 globally [5]. Global estimates for VLS are not readily available for children and adolescents, but cohort data among children and adolescents on ART at 143 sites in 30 countries show the suboptimal suppression rate of 64% at one year and 59% (38–91) at three years after ART initiation [6].

In 2016, WHO recommended multimonth dispensing (MMD) [7] as part of differentiated service delivery (DSD), with the goal of simplifying service delivery. In 2019, the U.S. President’s Emergency Plan for AIDS Relief (PEPFAR) actively recommended MMD and supported countries to extend dispensing intervals to up to six months [8]. Several studies from sub-Saharan Africa have shown that retention in care among patients enrolled in MMD is not inferior to that of the standard of care [9–12]. In addition, qualitative findings show that patients find the less frequent clinic visits more convenient and flexible [13]. Likewise, providers perceive benefits in MMD, including reduced burden on clients and on their own workload [14].

During the early months of the COVID-19 pandemic and at the peak of related restrictions, MMD and other non-facility DSD models were promoted by WHO [15,16], the United Nations Children’s Fund (UNICEF) [17], the Joint United Nations Programme on HIV/AIDS (UNAIDS) [18], the International AIDS Society [19], as well as being supported by donors such as the Global Fund [20] and PEPFAR [21–23] and national governments. However, countries were not able to implement stated policies in a timely manner due to supply chain challenges and limited stock.

Progress in rolling out MMD has been lagging among children and adolescents compared to adults. An internal 2020 PEPFAR MMD policy analysis in 21 supported countries showed that countries were limiting MMD to adults who were “stable on ART” [24], despite normative 2016 WHO [25] guidance recommending MMD for those receiving ART for at least one year, no current illnesses or pregnancy, a good understanding of lifelong adherence, and evidence of treatment success. Similarly, WHO recommends that children older than two years who have been receiving ART for more than one year and who fulfil other eligibility criteria should be eligible for MMD. As of June 2020, the adaptations to MMD policies in 16 PEPFAR-supported countries were associated with significantly accelerated growth in the proportion of clients on MMD for all populations, irrespective of age and dispensing interval [24].

Within this context, the Strengthening Integrated Delivery of HIV/AIDS Services (SIDHAS) project funded by PEPFAR through the United States Agency for International Development (USAID) and implemented by FHI 360 in the two high-burden states of Akwa Ibom and Cross River, Nigeria, collaborated with three USAID global technical assistance mechanisms—Meeting Targets and Maintaining Epidemic Control (EpiC), Reaching Impact, Saturation, and Epidemic Control (RISE), and Adolescents and Children HIV Incidence Reduction, Empowerment and Virus Elimination (ACHIEVE)—to expand MMD among children.

## Materials and methods

### Study design and objectives

We conducted a retrospective data analysis using routinely collected program data for CALHIV ages 18 years and younger. We excluded children under two years from the analysis, as MMD is not recommended for or routinely offered to children in that age group. The program data analyzed were from two populations—all CALHIV ages 2–18 receiving ART services at baseline and all those receiving ART services at endline—rather than a single cohort. We compared MMD coverage (primary objective), as well optimized regimen coverage, enrollment in community-based ART groups, viral load coverage, and viral load suppression (secondary objective), pre- and post-intervention (baseline and endline).

### Program setting and description

The SIDHAS project supported HIV care and treatment services in the two HIV high-burden states of Akwa Ibom and Cross River (adult HIV prevalence 4.8% and 1.8%, respectively [26]), including services to scale up MMD among CALHIV. Our retrospective analysis was limited to data from the period October 2019 through March 2021. Both states have hard-to-reach areas, and residents’ access to health facilities can be challenging. Implementing DSD models that reduce the frequency of clinical visits and ARV drug pickups and further decentralize drug distribution are critical for improving access to treatment for all populations and optimizing HIV program and clinical outcomes in these two states.

EpiC, a global technical assistance mechanism, and SIDHAS, the in-country implementer in Nigeria, collaborated to scale up MMD among CALHIV over the 18-month period through technical assistance to 36 “high-volume” facilities, defined as having five or more CALHIV currently on treatment. The high-volume sites selected for technical assistance in MMD scale-up ranged from primary health care centers to tertiary-level hospitals. At each site, the technical assistance provided by EpiC and SIDHAS was tailored to support MMD in the age groups where gaps were observed. This included the development of counseling messages about MMD for CALHIV and their caregivers which were culturally tailored and translated into local languages to address the needs of different ethnic groups in the two states.

As described in Table 1, the technical assistance involved conducting data analysis jointly with project and facility staff, conducting data reviews at the site level, identifying key bottlenecks, and designing actionable solutions, which included mentoring, line listing children and adolescents for enrollment, promoting the use of the pediatric regimen calculator to help clinicians select the correct regimens and dosages, and enrollment into DSD models.

**Table 1:** EpiC and SIDHAS technical assistance activities to scale up MMD among CALHIV.

Technical Assistance Activities	Description
Weekly and monthly data analysis and review	• Prioritized sites and age groups for site visits and mentoring activities through color-coded scoring based on set standards for VL and MMD • Prioritized sites with limited progress against the 80% MMD and 95% VLS PEPFAR benchmarks
Site visits to assess progress on the VL and MMD indicators (see right)	• Assessed the following indicators: • Proportion of CALHIV currently on treatment who had a documented VL test result among those who were eligible to receive such test
	• Proportion of CALHIV currently on treatment who were virally suppressed among those who had a documented VL test result
	• Proportion of CALHIV currently on treatment who were on 3–5-MMD and 6-MMD among those eligible to receive these services
Mentoring of health care providers in person and remotely (via phone, WhatsApp, and other virtual platforms)	• Supported staff on the following processes: • How to calculate and monitor the VL and MMD indicators
	• How to identify the CALHIV eligible for VL testing and MMD using the electronic medical record (EMR) system
	• Development of strategies to rapidly contact the CALHIV eligible for a VL test and/or MMD, collect the VL specimen, and transition them into MMD, including through the treatment supporter, peer or caregiver networks, phone calls, and home visits, along with the collection of VL specimens in the community where the CALHIV lived
	• Tailoring of counseling messages about MMD to CALHIV and their caregivers, including messages addressing weight monitoring, drug storage, adverse drug monitoring, and adherence to medication
Regular line listing of CALHIV eligible to receive VL testing and MMD at each site	• Developed a list of CALHIV eligible for VL test and for MMD through use of the EMR
Rollout of Pediatric Regimen Calculator	• Rolled out an Android application which informs health care providers on the correct regimen and dosage based on the child’s weight
Provision of optimized pediatric regimen	• Monthly stock monitoring of pediatric ART
	• Accurate reporting of the ART usage and stock and timely ordering of the necessary supply based on MMD projections
	• Medication redistribution across facilities in case of shortage
	• Provision of the following optimized ART regimens: Weight <20kg: ABC/3TC/LPV/r; weight 20–30kg: ABC/3TC/DTG; weight >30kg: tenofovir/3TC/DTG
Community ART distribution	• Provided ART refills within the community at the CALHIV and caregivers’ preferred venue, which was typically closer to their residence than the health facility of registration
Enrollment into pediatric and caregiver groups	• Enrolled clients in peer-led community- or facility-based groups where CALHIV and their caregivers received regular ART counseling and refills

### Data collection

Service data from the October–December 2019 quarter (baseline) through the January–March 2021 quarter (endline) were routinely collected by SIDHAS using standardized paper-based forms and then entered by data entry clerks into the electronic medical record (EMR) system—Lafiya Management information System (LAMIS). LAMIS is an open-source EMR system developed by FHI 360 Nigeria and used to manage client-level records of PLHIV enrolled in care. The system has features that facilitate client management, including generation of custom reports for PEPFAR and the Government of Nigeria, automated data quality assessment, and SMS messaging. The built-in validation rules in LAMIS include allowing only the record owner to enter or change specific fields in a record, a drop-down menu for data entry control, preformatted information fields (e.g., date, phone number), data-validation logic to prevent the entry of inconsistent information, and display of an error message if the information does not correspond to the preformatted limits.

De-identified data were then extracted from the LAMIS database. The extracted data included age, sex, and clinical information such as ART regimen, number of months of ART dispensed at the last refill, most recent VL test results, and enrollment in community ART groups.

### Data quality

All data were regularly validated through the project’s established processes for data quality assurance in which data were triangulated between the EMR and the paper tools, and identified gaps and outliers were reconciled, summarized, and reported daily and monthly. The data entry clerks reviewed the data on the paper-based forms for completeness and consistency before entering them into LAMIS. Built-in validation rules within LAMIS mitigated data entry and transcription errors. At the end of each day, a gap analyzer was run on LAMIS to identify data errors, which were then checked against the source documents and cleaned.

### Data analysis

For this study, the data were organized by age band: 2–4 years, 5–9 years, 10–14 years, and 15–18 years. We computed the number of CALHIV who were virally suppressed among those with a documented viral load (VL) test result within the past 12 months and the number of CALHIV on MMD among those currently on treatment. MMD was defined as receiving three or more months of antiretrovirals (ARVs) at a time per national guidelines. We disaggregated CALHIV on MMD into those being dispensed three to five months of ARVs at one time (3–5-MMD) and six or more months (6-MMD) dispensed. VLS was defined as having less than 1,000 copies, as per national and WHO guidelines [27].

We summarized MMD enrollment by computing the proportion of CALHIV on MMD and using a color-coded Excel form in which red indicated <70%, yellow 70%–79%, and green ≥80%. VLS was similarly categorized, with red indicating <70%, yellow 70%–94%, and green ≥95%. Descriptive statistics were used to summarize the characteristics of CALHIV on MMD and those not on MMD.

We summarized the number of CALHIV on optimized regimens based on 2018 WHO recommendations, included using abacavir and lamivudine (ABC/3TC) and either dolutegravir (DTG) or lopinavir/ritonavir (LPV/r) as first-line regimens in infants and children weighing <20 kg; ABC/3TC and DTG50 mg in children 20–≤30 kg; and the fixed-dose combination of tenofovir, lamivudine, and dolutegravir (TLD) in adolescents ≥30 kg or more.

In addition, we determined the proportion of CALHIV enrolled into the various DSD models offered by the project simultaneous with MMD enrollment. We computed the number of CALHIV enrolled in community-based ART refill clubs led by health workers, client-led groups, and facility-based adolescent refill clubs, the three models implemented in the region.

### Ethical considerations

The study received ethical approval from FHI 360’s Protection of Human Subjects Committee (approval number: 1759454–1). Informed consent requirements were waived because data from the EMRs included in the study analysis were fully anonymized, and client identifiers were excluded in the abstracted data. The authors did not have access to individual identifiers at any time.

## Results

At the end of the October–December 2019 quarter, SIDHAS was supporting 155 health facilities where 65,946 adults and 2,563 children ages 2–18 years were on ART. Thirty-nine of these were high-volume facilities and received technical assistance. By the end of March 2021, the number of children on ART had increased to 4,425.

### Progress on MMD

**Fig 1:**
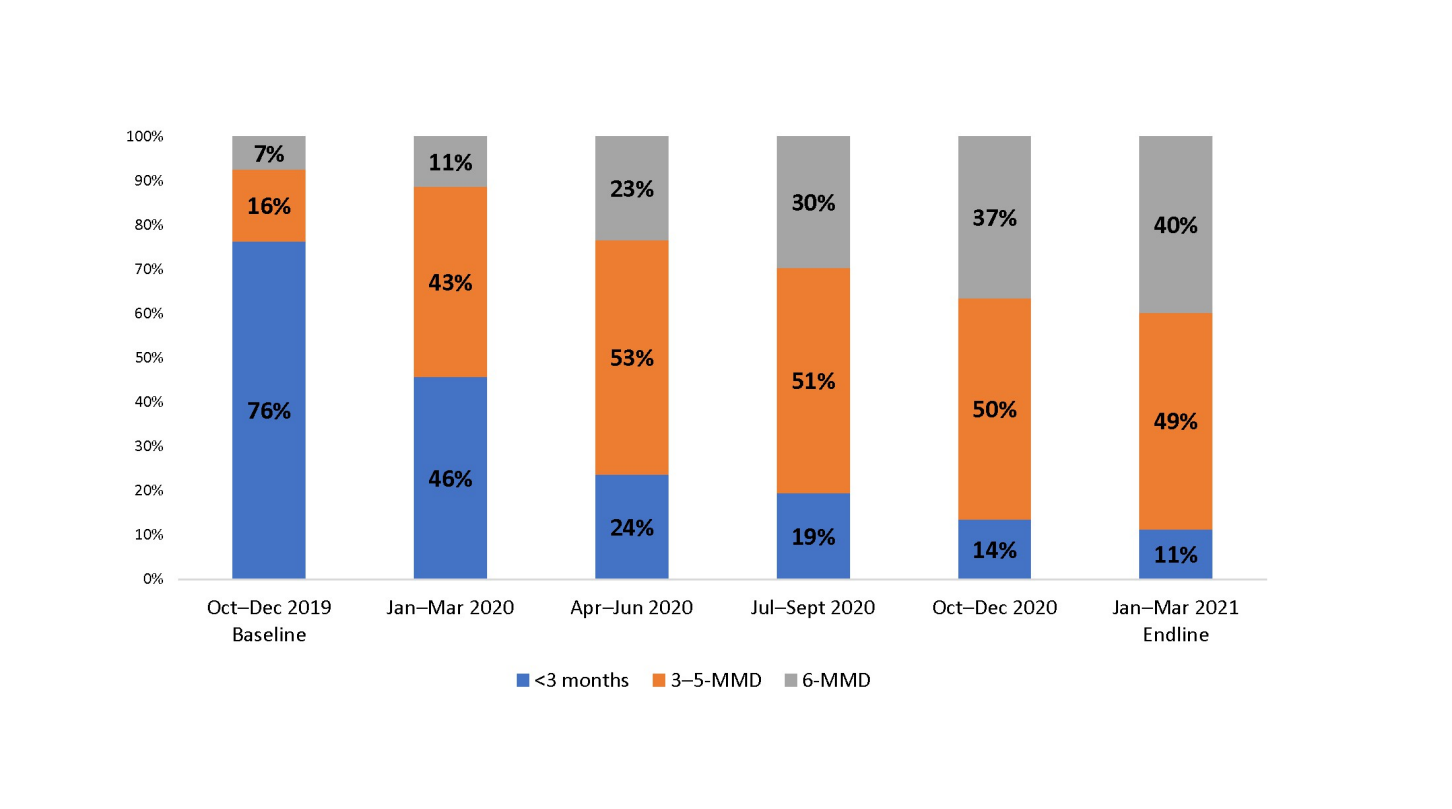
Proportion of CALHIV ages 2–18 years receiving MMD among those currently on ART at 36 SIDHAS-supported sites in Akwa Ibom and Cross River states, Nigeria, by quarter (October 2019–March 2021).

Fig 1 shows quarter-by-quarter progress in scale-up of MMD among CALHIV from baseline in the October–December 2019 quarter to endline in the January–March 2021 quarter. Over the implementation period, the proportion of CALHIV receiving MMD increased from 23% to 88%. As of March 2021, about half of the CALHIV were receiving 3–5-MMD, and almost 40% were receiving 6-MMD. Scale-up of 6-MMD increased progressively each quarter.

**Fig 2:**
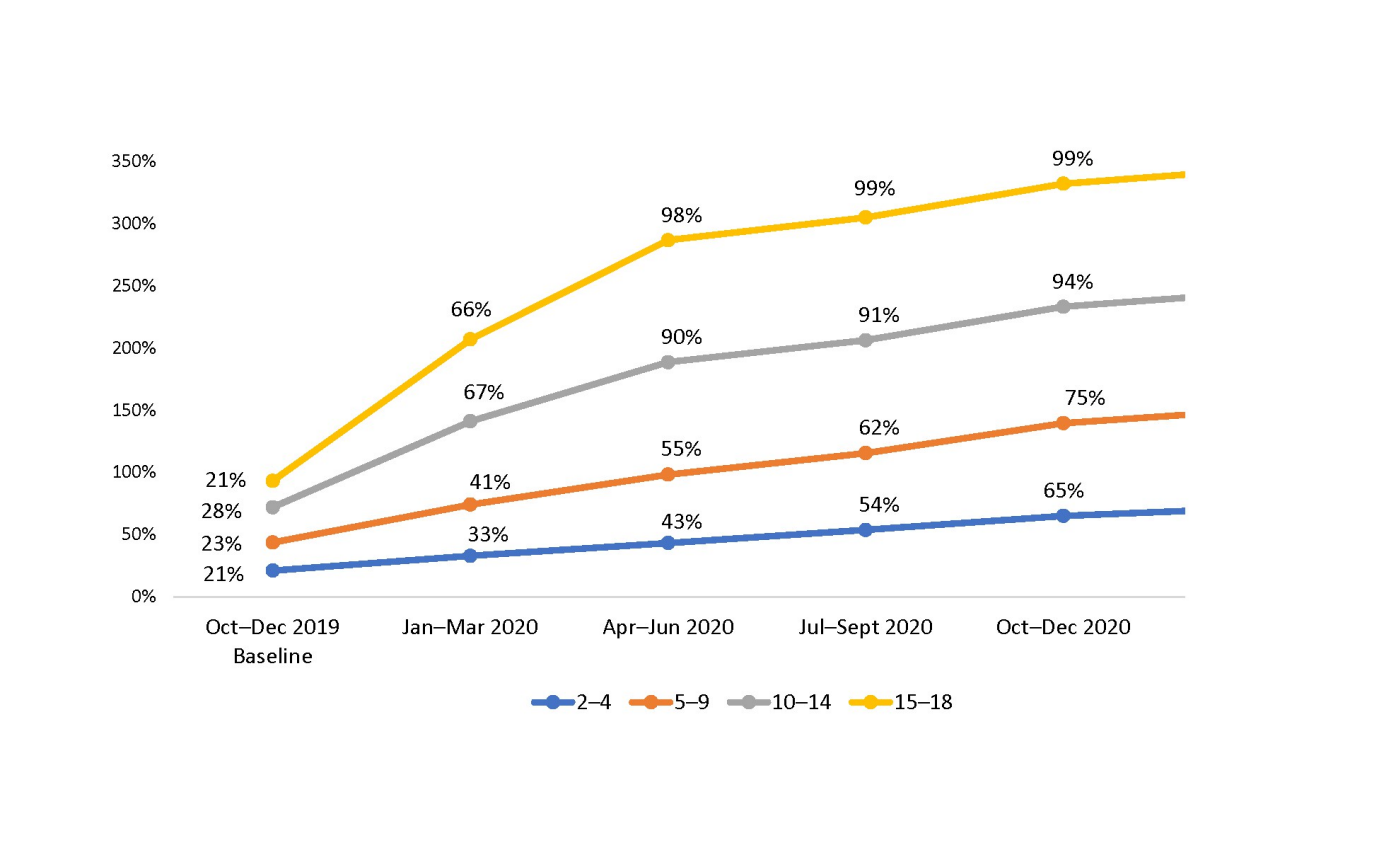
Number and proportion of CALHIV on MMD among those currently on ART at 36 SIDHAS-supported sites, by age group and quarter (October 2019–March 2021).

When disaggregated by age group, in October–December 2019, about one-quarter to one-third of the CALHIV in each age group were receiving MMD; in January–March 2020, 99% of adolescents (ages 15–18 years), 94% of 10–14-year-olds, 79% of 5–9-year-olds, and 71% of 2–4-year-olds were on MMD (Fig 2 and Table 2).

**Table 2:** Number of CALHIV currently on ART at 36 SIDHAS-supported sites, by age group and quarter (October 2019–March 2021).

Age Group, Years	CALHIV Currently on ART					
	Oct–Dec 2019 Baseline	Jan–Mar 2020	Apr–Jun 2020	Jul–Sep 2020	Oct–Dec 2020	Jan–Mar 2021 Endline
2–4	391	539	560	622	651	655
5–9	787	1,009	1,034	1,079	1,070	1,148
10–14	726	895	929	1,016	1,059	1,124
15–18	659	1,150	1,257	1,449	1,521	1,498

**Fig 3:**
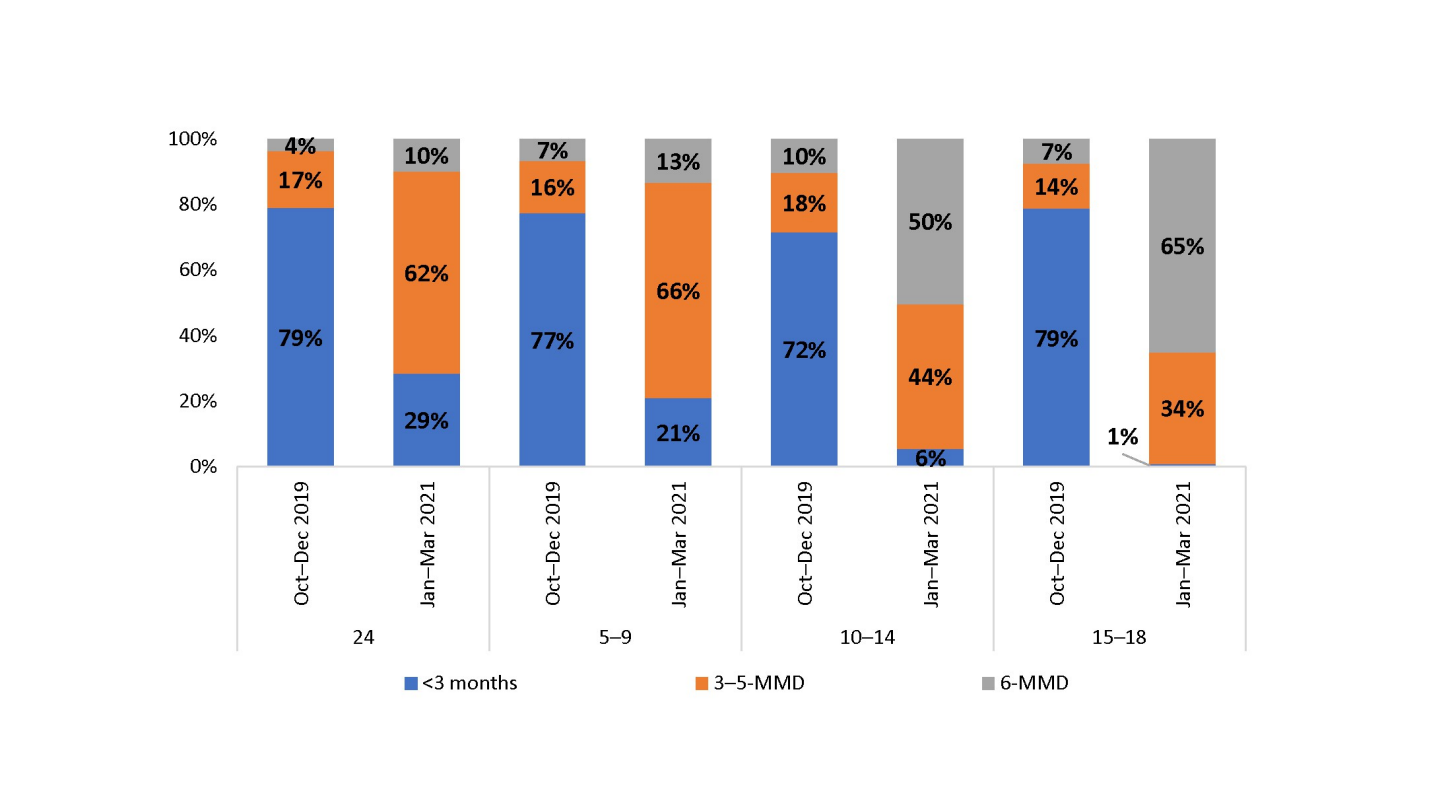
Proportion of CALHIV on MMD among those currently on ART at 36 SIDHAS-supported sites, by ARV supply and age group (October–December 2019 vs. January–March 2021).

When disaggregated by age group and comparing baseline to endline, the increase in uptake of 6-MMD was greater among children and adolescents 10 years and older, while the uptake of 3–5-MMD was greater among those ages 2–9 years (Fig 3).

When sites were assessed using a color-coded performance matrix, the proportion of sites scoring red (<70% of children on MMD) for CALHIV ages 2–18 decreased from 100% to 28% from baseline to endline. The gaps in meeting the benchmark were mostly in ages nine years and younger.

### Progress on viral load testing coverage

Because of concerns among health providers that MMD would interfer with collection of VL samples, we assessed the proportion of CALHIV who had a documented VL test result within the past 12 months (VL testing coverage) (Table 3). Overall, we did not observe a decline in the proportion of children with a documented VL test result. Instead, we observed a slight increase among children ages 2–4 and 5–9 years, whose VL testing coverage increased from 75% to 84% and 86% to 91%, respectively, between baseline and endline.

**Table 3:** Proportion of CALHIV at 36 SIDHAS-supported sites who had a documented VL test within the past 12 months among those currently on treatment for at least six months, by age group and quarter (April 2019–March 2021).

	Currently on Treatment							VL Testing Coverage					
Age Group, Years	Apr–Jun 2019	Jul–Sept 2019	Jan–Mar 2020	Apr–Jun 2020	Jul–Sept 2020	Oct–Dec 2020	Jan–Mar 2021	Oct–Dec 2019 Baseline	Jan–Mar 2020	Apr–Jun 2020	Jul–Sept 2020	Oct–Dec 2020	Jan–Mar 2021 Endline
2–4	324	432	391	539	560	622	651	75%	69%	81%	63%	78%	84%
5–9	680	824	787	1,009	1,034	1,079	1,070	86%	87%	89%	72%	85%	91%
10–14	546	747	726	895	929	1,016	1,059	103%	91%	93%	80%	98%	97%
15–18	486	739	659	1,150	1,257	1,449	1,521	90%	92%	118%	80%	93%	89%
2–18	2,036	2,742	2,563	3,593	3,780	4,166	4,301	90%	86%	96%	75%	90%	91%

### Progress on viral load suppression

**Fig 4:**
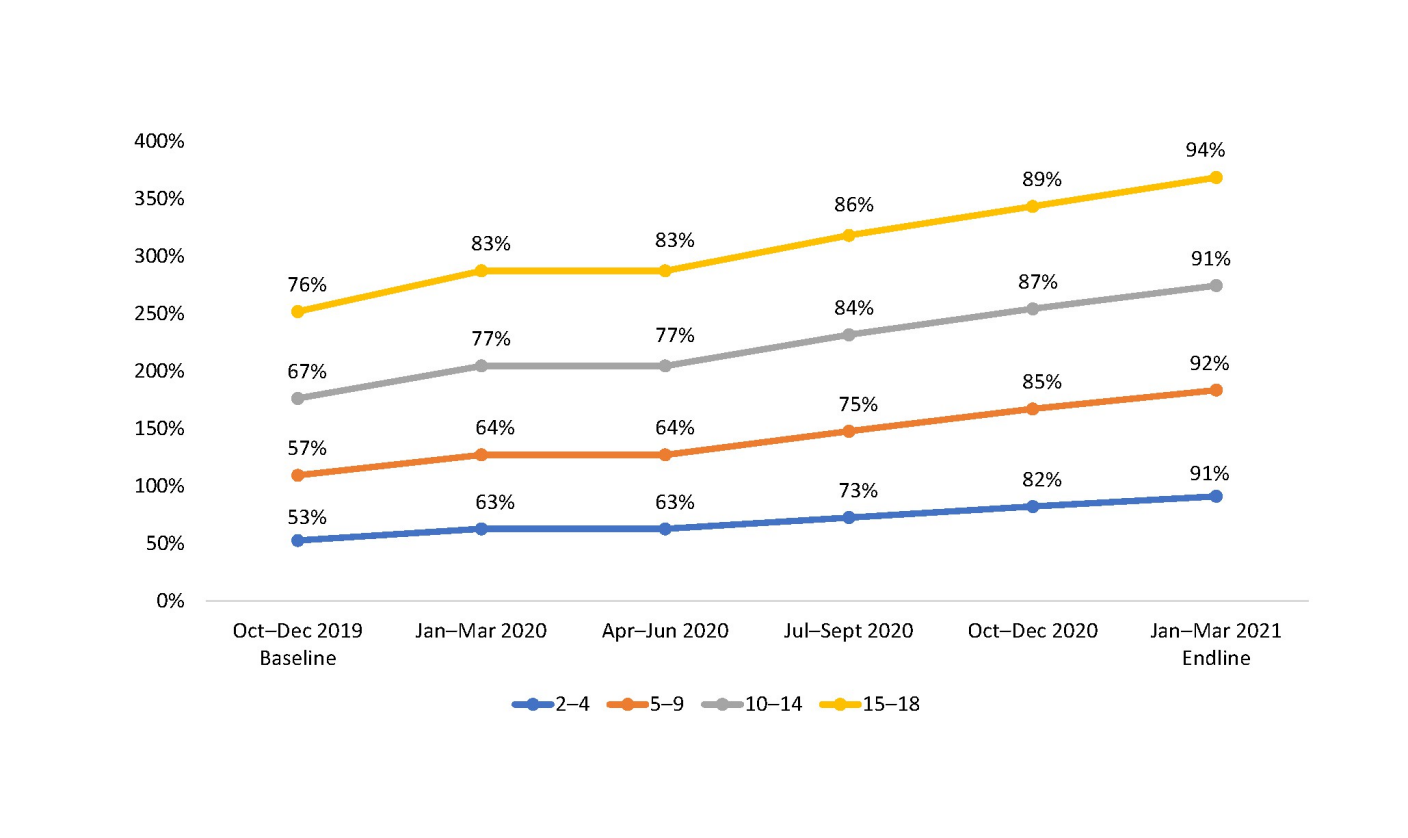
Proportion of virally suppressed CALHIV among those with a VL test result within the past 12 months at 36 SIDHAS-supported sites, by age group and quarter (October 2019–March 2021).

Fig 4 show the proportion of CALHIV who were virally suppressed during the implementation period among those who had a documented VL test within the previous 12 months (Table 4). Overall, the proportion of CALHIV who were virally suppressed increased from 64% in October–December 2019 to 92% in January–March 2021. The rate of increase in the proportion of virally suppressed CALHIV was observed across all age groups but was highest among children ages 2–4 years and 5–9 years (from 53% and 57%, respectively, in October–December 2019, to 91% and 92%, respectively, in January–March 2021) and lowest among those ages 15–18 years (76% in October–December 2019, to 94% in January–March 2021).

**Table 4:** Number of CALHIV with a VL test result within the past 12 months at 36 SIDHAS-supported sites, by age group and quarter (October 2019–March 2021).

Age Group, Years	CALHIV with a Documented VL Test within the Past 12 Months					
	Oct–Dec 2019 Baseline	Jan–Mar 2020	Apr–Jun 2020	Jul–Sept 2020	Oct–Dec 2020	Jan–Mar 2021 Endline
2–4	242	297	316	342	439	525
5–9	584	716	702	730	878	986
10–14	565	677	675	716	908	982
15–18	438	677	775	919	1,167	1,293

When assessed based on the color-coded performance matrix for VLS from baseline to endline, the proportion of sites scoring red (<70% of CALHIV virally suppressed) decreased from 79% to 0%.

### Use of optimized regimens

**Fig 5:**
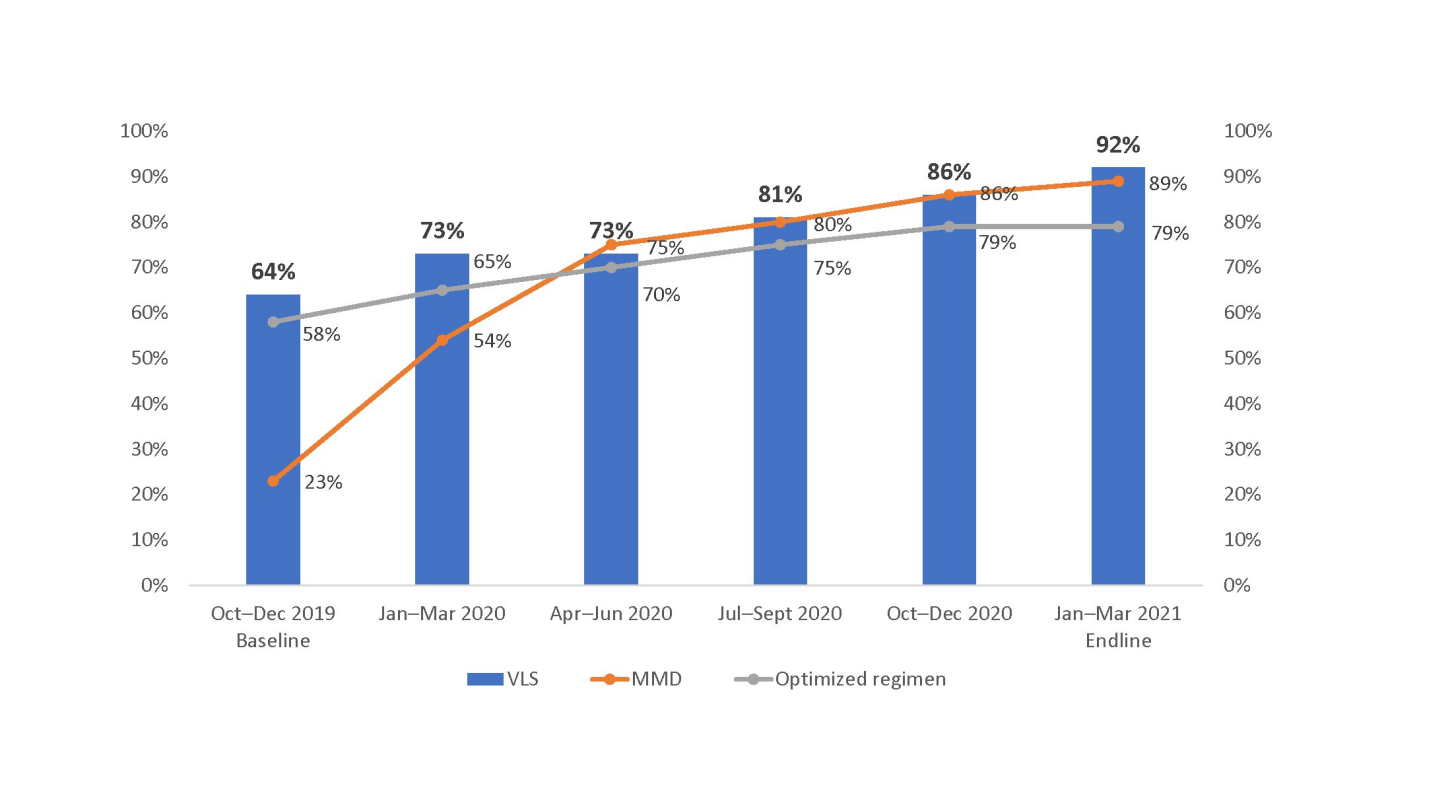
Proportion of CALHIV ages 2–18 years currently on ART, on optimized pediatric regimens, on MMD, and virally suppressed at 36 SIDHAS-supported sites, by quarter (October 2019–March 2021).

Over the implementation period, we observed an increase in the proportion of CALHIV on optimized regimens and those on MMD and virally suppressed (Fig 5).

**Fig 6:**
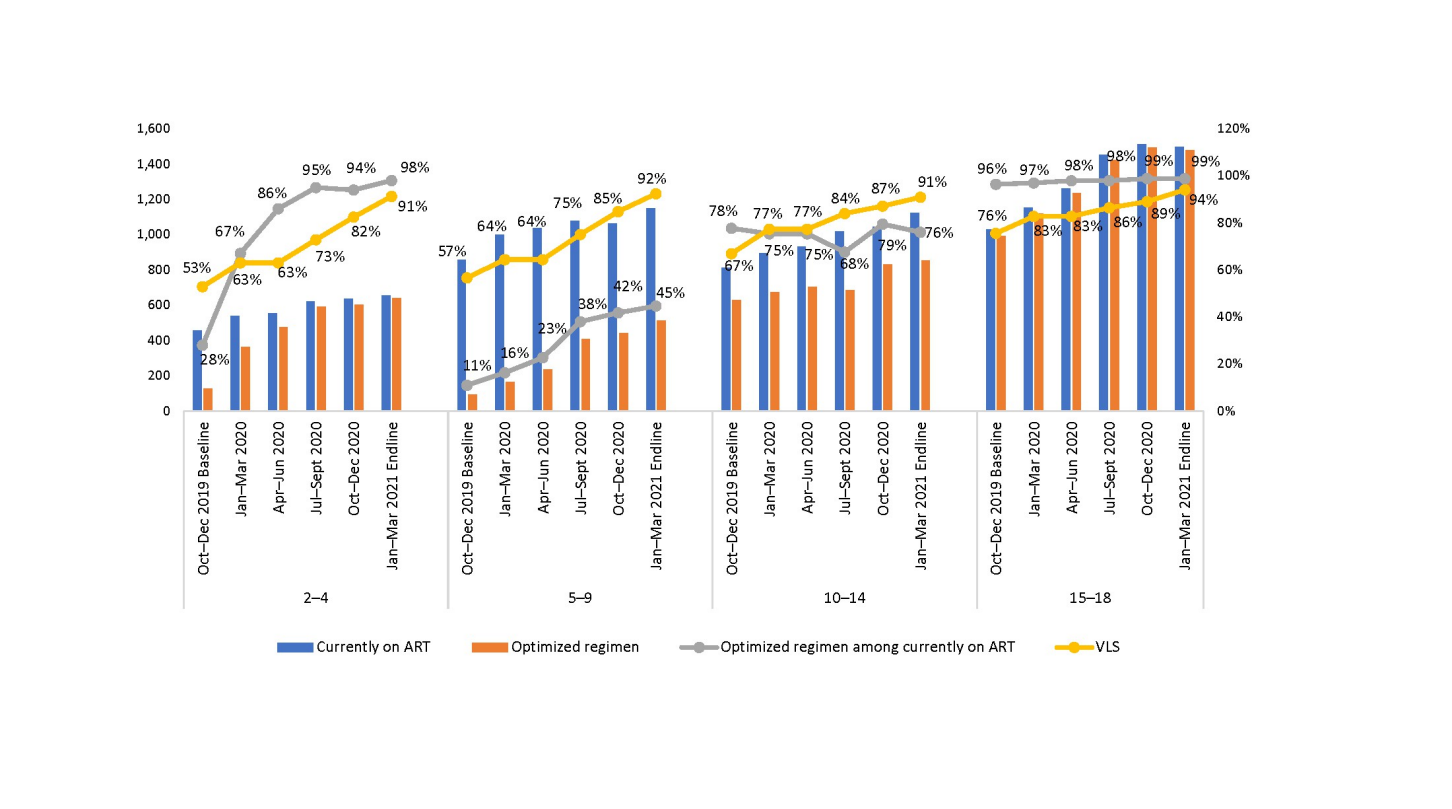
Proportion of CALHIV currently on ART, on optimized pediatric regimens, and virally suppressed at 36 SIDHAS-supported sites, by age group and quarter (October 2019–March 2021).

When analyzed by age group (Fig 6), both VLS and transition to optimized pediatric regimens progressively increased. At endline, close to 90% of the CALHIV were virally suppressed. However, despite only 42% of CLHIV ages 5–9 years being on optimized regimens, VLS reached 92% at endline.

**Fig 7:**
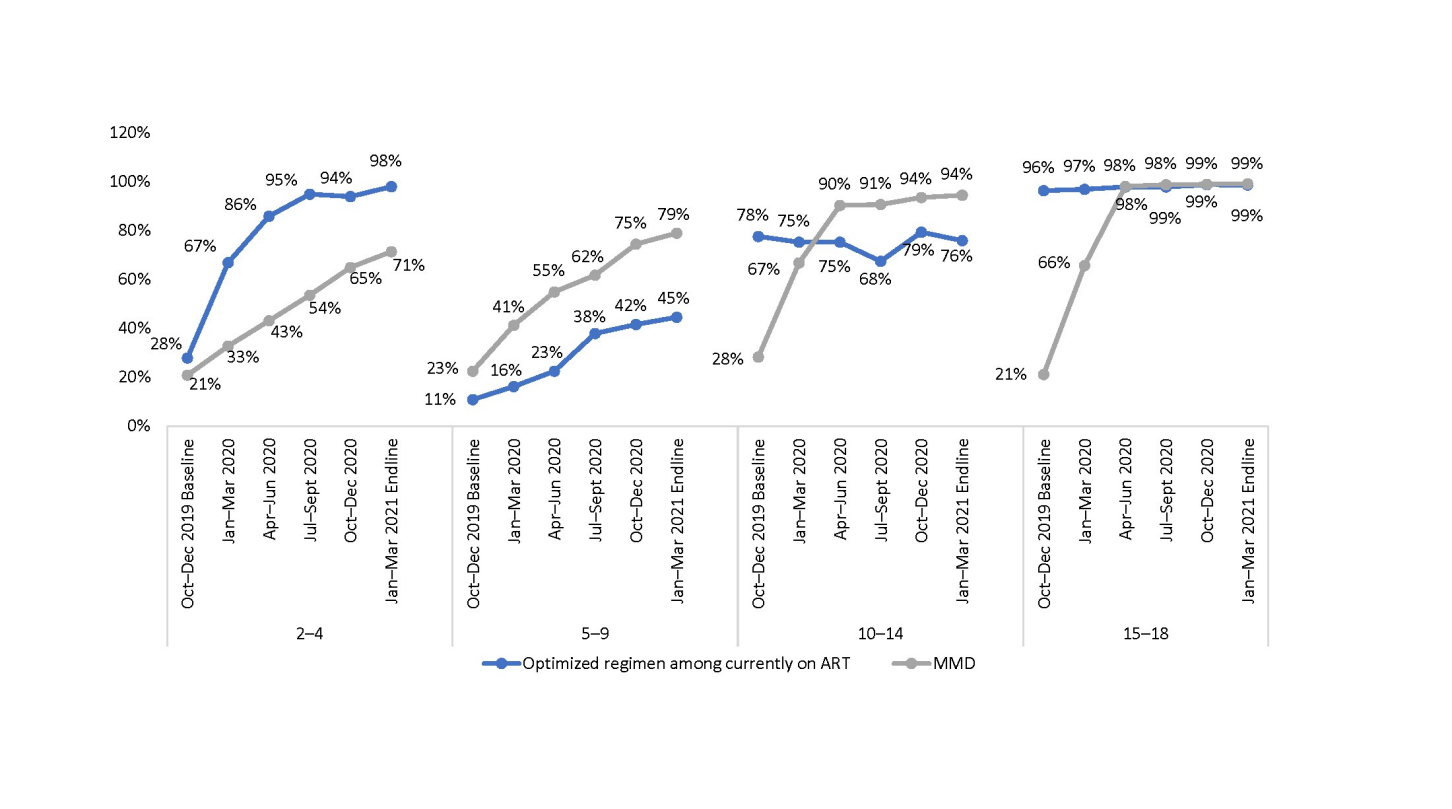
Proportion of CALHIV currently on ART on optimized pediatric regimens and on MMD at 36 SIDHAS-supported sites, by age group and quarter (October 2019–March 2021).

When disaggregated by age group (Fig 7), at baseline the proportion of CALHIV on MMD was higher than those on optimized regimens for ages 10–18 years, while for ages 2–9 years, both proportions were similarly low. Virtually all ALHIV ages 15–18 years were on optimized regimens and MMD at endline.

Table 5 shows the stock and consumption status of different pediatric regimens. In January–March 2021, all nonoptimized regimens were no longer in stock.

**Table 5:** Months of stock by pediatric ARV medication at 36 SIDHAS-supported sites (October–December 2019 vs. January–March 2021).

S/N	ARV Medication	October–December 2019			January–March 2021		
		Stock on Hand	Average Monthly Consumption	Months of Stock	Stock on Hand	Average Monthly Consumption	Months of Stock
1	DTG 50 mg	155	450	0.3	2,402	1,190	2.0
2	ABC|3TC 120 mg|60 mg	593	1,405	0.4	10,196	3,978	2.6
3	LPV|r 100 mg|25 mg	197	117	1.7	4,616	1,499	3.1
4	LPV|r 40 mg|10 mg	196	145	1.4	1,720	828	2.1
5	AZT|3TC 60 mg|30 mg	126	25	5.0	0	0	0
6	EFV 200 mg	73	32	2.3	0	0	0
7	AZT|3TC|NVP 60 mg|30 mg|50 mg	2,814	377	7.5	6	9	0.7

### Other interventions to optimize the outcomes for CALHIV

**Fig 8:**
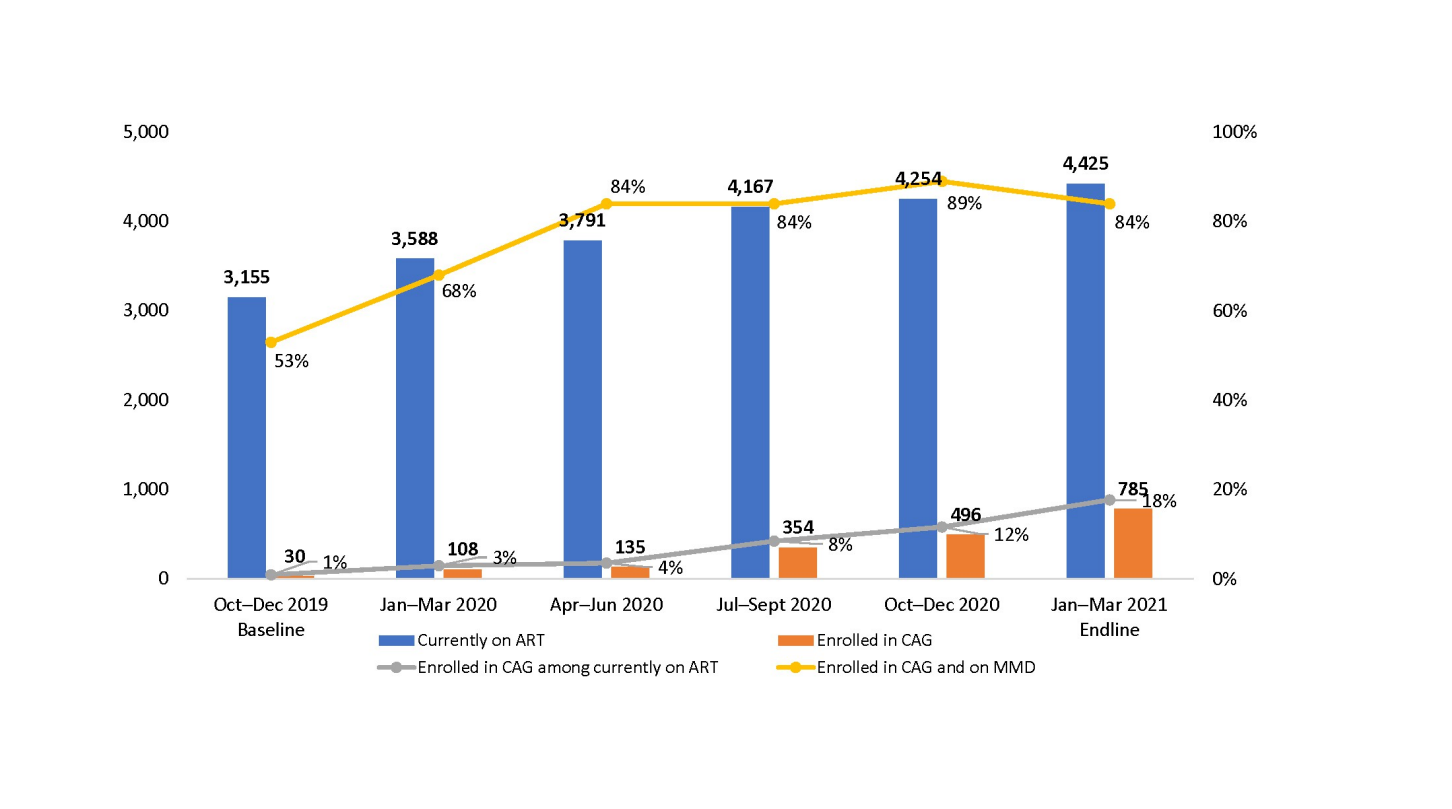
Proportion of CALHIV ages 2–18 years currently on ART, enrolled in CAGs, and on MMD at 36 SIDHAS-supported sites, by quarter (October 2019–March 2021).

Even though a relatively small proportion of CALHIV currently on ART were enrolled in community-based ART groups (CAGs) (18%), most of those enrolled were on MMD (84%) as of March 2021 (Fig 8).

### Follow-up after endline

**Fig 9:**
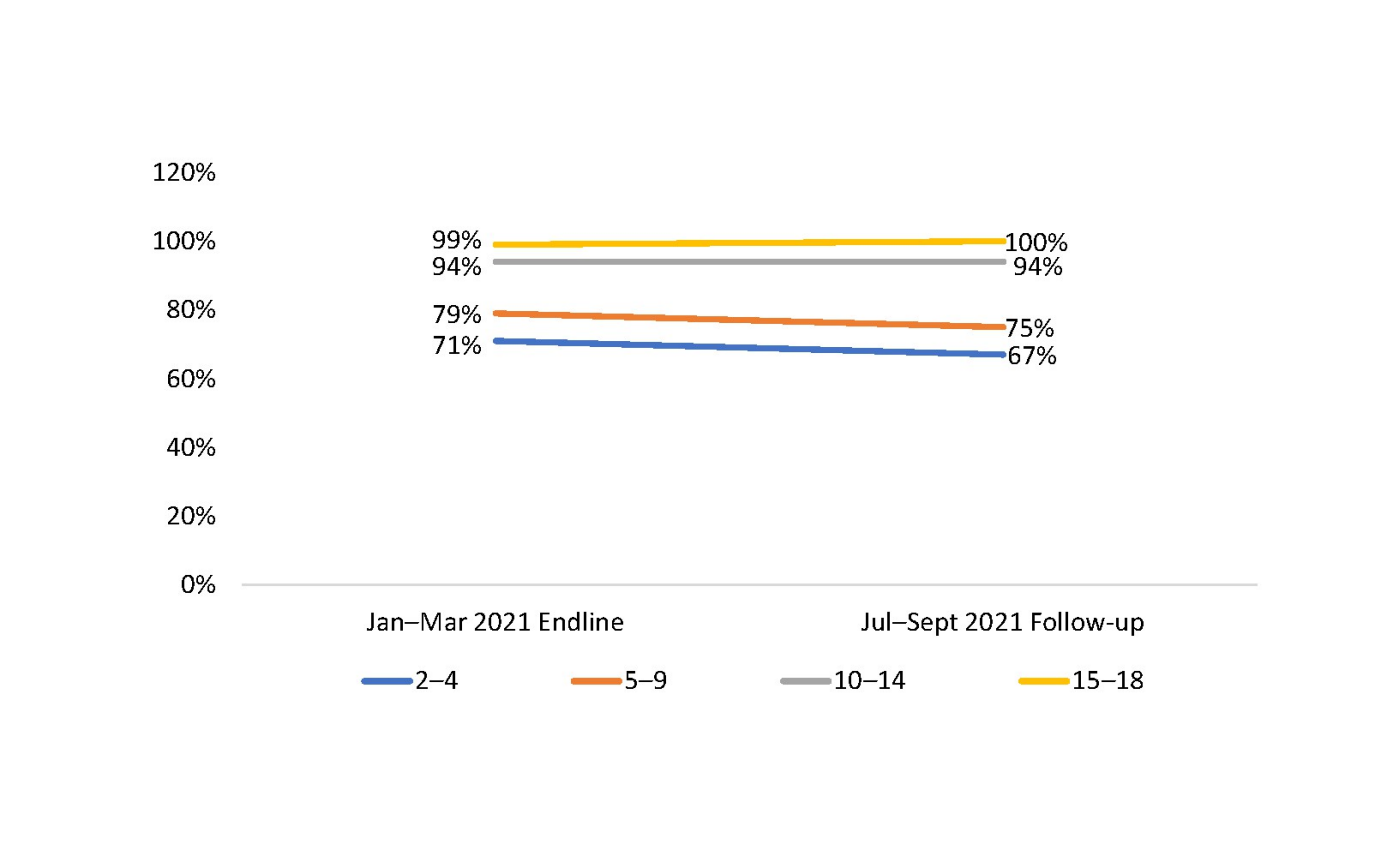
Proportion of CALHIV on MMD among those currently on ART at 36 SIDHAS-supported sites, by age group January–March 2021 (endline) vs. June–September 2021.

By September 2021, six months after the end of active technical assistance, the proportion of children on MMD remained high (Fig 9).

## Discussion

In this study, the MMD coverage among CALHIV ages 2–18 years increased almost four-fold and exceeded the 80% PEPFAR global benchmark for PLHIV following technical assistance provided to the sites by SIDHAS and EpiC—namely, site-level granular data analysis, stakeholder consultation, co-creating solutions to key bottlenecks, and designing actionable solutions. However, low stocks of ARV medications limited the proportion of children receiving six months of ARV refills.

Our encouraging results were likely due to several interventions delivered concurrently as part of our technical assistance. One was the use of a color-coded performance matrix to score site performance for MMD and VLS by age group enabled tailoring of technical assistance to address discrete gaps. The simple visual tool also allowed specific sites to identify weaknesses and co-create solutions with technical assistance teams. Technical assistance teams were able to demonstrate the importance of a granular data-driven approach and use the indicators to guide their actions.

A second contributing intervention was regular line listing of individual CALHIV to identify those eligible for VL testing and MMD enrollment and ensure they were on appropriate regimens and dosages, which encouraged a culture of data use for decision-making. Improved VL testing coverage through active line listing of clients has shown to lead toward better clinical outcomes and increased retention in care [28].

The higher rate of increase of MMD coverage in children ages 2–9 years observed in our study was likely driven by a lower proportion on MMD at baseline compared to the oldest age group, many of whom were already on MMD. However, at endline, children younger than nine years were less likely to be on MMD than those older than nine. The proportion was especially lower in children ages 2–4 years. These findings suggest a possible lack of confidence among the health care providers in offering MMD to younger age groups.

We were unable to achieve the projected rates of 6-MMD, mainly because of challenges related to supply chain. It is also likely that many providers were uncomfortable with and found it difficult to offer MMD to children in general for fear that dosage adjustment milestones would be missed. Given that only three ART dose adjustments are anticipated between the ages of one to seven years [29], these fears are unfounded, and more efforts should be put into addressing clinician hesitancy as well as supply-chain bottlenecks.

We documented higher rates of VL testing coverage and VLS than reported in similar settings. In-depth qualitative surveys from India, Kenya, Malawi, South Africa, and Zimbabwe found that only a small proportion of patients on ART received a VL test in one year [30]. A systematic review and meta-analysis found viral suppression rates of 60%–75% over the past 15 years in children living in low middle-income countries [31]. In this setting, therefore, VL testing and suppression were not impeded by MMD scale-up and vice versa.

An additional factor affecting our results was that the study was conducted during the COVID-19 pandemic, when MMD was a necessity to ensure treatment continuity. Indeed, during the pandemic, the MMD eligibility criteria in Nigeria and other countries were expanded, and the requirement to be virally suppressed to qualify for MMD was waived [24]. These policy changes may explain why the number of CALHIV on MMD was higher than those who were virally suppressed. These policy changes enacted during the COVID-19 pandemic were developed to assure treatment continuity and have resulted in substantial enrollment into DDD and MMD.

At the end of our multipronged intervention, more than 90% of the CALHIV in each age group were virally suppressed, with the highest increase observed in children ages 2–9 years. This finding is encouraging, and lessons learned from our experience could help other programs improve outcomes for children.

The regular stock monitoring and availability of the appropriate pediatric ART regimens facilitated the transition to MMD, along with the use of technology (e.g., virtual platforms for remote mentoring, the Android app for calculating the pediatric dosage), and supported the transition to optimized regimens and correct dosing schedules—as well as the pace of MMD scale-up. We observed a marked increase in the proportion of children on optimized regimens and the eventual reduction in legacy regimens in stock at the sites.

Enrollment in community-based ART groups and refills brings services closer to CALHIV and their caregivers and could also serve as a platform to roll out MMD. Similar observations have been reported in other countries [32]. Distributing ARVs in the community likely contributed to a modest number of children receiving their ARVs at more convenient locations. These community-based interventions leveraged existing infrastructure, including local government spaces, after-hours at schools, community-based organization facilities, and private residences. We also supported makeshift outreach clinics for the purpose of offering ART and VL testing within the communities. The mapping and the availability of such communal spaces and the funding of mobile outreach services are critical enablers of community-based interventions.

Lastly, we were able to demonstrate the sustainability of our technical assistance as the proportion of children and adolescents on 6-MMD after the end of active support remained high, suggesting that the interventions rolled out over the course of the intensified technical assistance phase could be effective long term.

Our study had several limitations. First, the data used were routinely collected aggregated data. Second, because of the quantitative nature of the assessment, we could not identify more detailed contextual factors affecting MMD uptake. For example, the providers were uncomfortable offering MMD to children in general for fear that dosage adjustment milestones would be missed, but we did not explore caregivers’ concerns regarding this issue, and we did identify any literature that dealt specifically with caregiver concerns about MMD for the children in their care. Third, because we limited our assessment and support to high-volume targets, these findings are not representative of all children; nonetheless, to our knowledge, this is the first study describing MMD progress among CALHIV in Africa.

## Conclusions

MMD among CALHIV is feasible. The provision of data-driven technical assistance and the employment of other interventions such as pediatric ARV stock monitoring, use of a pediatric regimen application, and the rollout of CAGs supported the transition of CALHIV on MMD across all age groups and contributed to achieving high rates of viral suppression. Although the proportion of CALHIV with a documented VL test result, VLS, on MMD, and on optimized regimens increased over time, more targeted interventions are needed for younger children 2–9 years, among whom the benchmarks were not achieved. As COVID-19-related emergency directives played a role in scaling up MMD, efforts are needed to ensure that policy and guidelines are formalized if epidemic control among CALHIV is to be achieved.

## Supporting information

S1 File (XLSX)
